# A theory of migration: the aspirations-capabilities framework

**DOI:** 10.1186/s40878-020-00210-4

**Published:** 2021-02-24

**Authors:** Hein de Haas

**Affiliations:** University of Amsterdam, Amsterdam, Netherlands

**Keywords:** Migration, Migration theory, Social theory, Development, Social transformation

## Abstract

This paper elaborates an aspirations–capabilities framework to advance our understanding of human mobility as an intrinsic part of broader processes of social change. In order to achieve a more meaningful understanding of agency and structure in migration processes, this framework conceptualises migration as a function of aspirations and capabilities to migrate within given sets of perceived geographical opportunity structures. It distinguishes between the instrumental (means-to-an-end) and intrinsic (directly wellbeing-affecting) dimensions of human mobility. This yields a vision in which moving and staying are seen as complementary manifestations of migratory agency and in which human mobility is defined as people’s capability to choose where to live, including the option to stay, rather than as the act of moving or migrating itself. Drawing on Berlin’s concepts of positive and negative liberty (as manifestations of the widely varying structural conditions under which migration occurs) this paper conceptualises how macro-structural change shapes people’s migratory aspirations and capabilities. The resulting framework helps to understand the complex and often counter-intuitive ways in which processes of social transformation and ‘development’ shape patterns of migration and enable us to integrate the analysis of almost all forms of migratory mobility within one meta-conceptual framework.

## Introduction

Migration theory has been at an impasse for several decades (see Arango 2000; de Haas 2010a; Massey et al. 1993; Massey 2019; Skeldon 2012). The field of migration studies has remained a surprisingly under-theorised field of social inquiry. This is unfortunate, as we can only develop a richer understanding of migration processes if we do not conceptually separate them from broader processes of social change of which they are a constituent part. Much thinking on migration remain implicitly or explicitly based on simplistic push–pull models or neo-classical individual income (or ‘utility’) maximising assumptions, despite their manifest inability to explain real-world patterns and processes of migration. Although prior migration theories have been rightfully criticised for their unrealistic assumptions, researchers have generally been better at debunking such theories than at coming up with viable theoretical alternatives.

To overcome this impasse and to advance our understanding of migration processes as an intrinsic part of broader processes of social change and ‘development’, this paper elaborates a theoretical framework that conceptualises migration as a *function of people’s capabilities and aspirations to migrate within given sets of perceived geographical opportunity structures*. Applying Sen’s (1999) capabilities framework to migration, this paper defines *human mobility* as people’s capability (freedom) to choose where to live – including the option to stay – instead of a more or less automated, passive and ‘cause-and-effect’ response to a set of static push and pull factors. The paper draws on Berlin’s (1969) concepts of positive and negative liberty to theorise the complex and non-linear ways in which macro-structural change can shape migration aspirations and capabilities, as well as to define new, theory-derived categories of human mobility and migration.

## Migration theory: what is the problem?

Migration studies is an under-theorised field of social-scientific inquiry, in which the recent trend has been one of theoretical regression rather than progress. Earlier contributions to the field – such as Lee’s (1966) theory of migration, Mabogunje’s (1970) migration systems theory, Zelinsky’s (1971) mobility transition theory, Skeldon's (1990) work on migration transitions, Harris and Todaro’s (1970) neo-classical migration theory, Piore’s (1979) dual labour-market theory, Stark’s (1978, 1991) new economics of labour migration and Massey’s (1990) cumulative causation theory – all tried to come up with generalised understandings of migration phenomena. With the exception of a few authors (Carling 2002; Faist 2000; Hatton and Williamson 1998; Skeldon 1997), in more recent decades the systematic theorisation of migration processes has been largely abandoned (see Skeldon 2012). In their seminal overview of migration theories, Massey and his colleagues (Massey et al. 1993, p. 432) concluded that much thinking on migration ‘remains mired in nineteenth-century concepts, models, and assumptions’. Unfortunately, not much has changed since then.

This state of theoretical underdevelopment strongly contrasts with the huge increase in the number of empirical studies on migration. The lack of systematic theorising hampers our ability to meaningfully interpret empirical ‘facts’, to understand how macro-structural factors shape migration processes as well as to explain the huge diversity in migration experiences across different ethnic, gender, skill and class groups. Particularly since the rise of ‘postmodern’ social science in the 1970s and 1980s, big-picture migration theory-making has been largely abandoned. In reaction to the critique on ‘grand theory’ as well as the state-bias and ‘methodological nationalism’ (Wimmer and Glick Schiller 2002) inherent in much (policy-driven) migration research, recent work, particularly by anthropologists and sociologists, has focused on studying and conceptualising the (transnational, multicultural, diasporic, creolised) lives, identities and experiences of migrants from an ‘emic’ perspective.

Notwithstanding the considerable merits of such research, this has unfortunately coincided with an increasing gap between sociologists, anthropologists and also geographers conducting qualitative, interpretative micro-studies on migrants’ experiences on the one hand and the branches of economics, sociology and demography that have increasingly focused on quantitative regression analysis to examine the ‘causes’ and ‘impacts’ of migration largely along the (implicit or explicit) lines of the ‘push–pull’ model. While qualitative researchers often seem to have rejected the idea of explanatory migration theories altogether as naïvely positivist, the theoretical veneer of quantitative approaches has also remained extremely thin, as they do generally not go beyond an (often implicit) functionalist ‘push–pull’ perspective, according to which migrants are actors seeking to maximise income or ‘utility’. Both qualitative and quantitative approaches have failed to adequately capture the vital role of difficult-to-quantify structural factors such as inequality, power and states in shaping migration processes, or to develop a meaningful idea of human agency beyond the voluntaristic assumptions of neo-classical models or the portrayal of migrants as more or less passive victims of capitalist forces, as is common in historical-structural theories.

## The ‘migration is too complex’ fallacy

The central problem in migration research is the absence of a central body of theories that summarises, generalises and systematises the accumulated insights of a vast amount of empirical research, that can serve as a common frame of reference within which to examine, interpret, understand and explain 'facts' and ‘findings’ from various disciplinary and paradigmatic perspectives, and that can guide future research. Several factors contribute to this lack of progress in our generalised understanding of migration, including:

- the ‘receiving country bias’ and the concomitant ignorance of the causes, consequences and experiences of migration from an origin-area perspective, leading to one-sided, biased understandings of migration;
- the dominance of government perspectives, ‘methodological nationalism’ (see Wimmer and Glick Schiller 2002) and the related tendency to uncritically adopt state categories to classify migrants and migration, which often sustain distorted, ideological views on migration;
- disciplinary and methodological divides, particularly between quantitative (positivist) and qualitative (interpretative) approaches;
- the divide between the study of ‘forced’ and that of ‘voluntary’ migration; and
- the divide between the study of international and that of internal migration.

Researchers have frequently argued that a comprehensive or universal migration theory will never arise because migration is too complex and diverse a phenomenon (see Castles and Miller 2009; Salt 1987). However, this argument is not convincing for two main reasons. First, it would be misleading to suggest that the goal of social theory is to develop all-explaining, *universal* theories because social phenomena always need to be understood within the specific historical and social contexts in which they occur and can therefore never be captured by a simple set of formulas, ‘laws’, models or regression equations. Second, complexity can never be a reason to abandon efforts to build better social theories. After all, social phenomena are complex by nature and complexity has not stood in the way of theoretical advancement in other fields of social inquiry. In fact, we need to turn the argument upside down: the complexity which is so characteristic of social processes is the very reason why we need social theories, as they help us to make sense of and to discern patterns amidst the sometimes dazzling diversity – and the apparently random, chaotic and non-systemic nature – of human experiences and social interactions. In other words, social theories help us to see the wood for the (empirical) trees.

Importantly, the notion of complexity does not imply that social phenomena and social processes are chaotic or devoid of regularities, patterns or structure. Rather, complexity implies that they consist of many parts in elaborate, multi-layered arrangements. From a micro-perspective, the diversity of migration experiences may seem bewildering but, once we start to zoom out, regularities and patterns tend to emerge. This reflects the very purpose of social theory: to discern patterns in order to make sense of what is happening around us. For instance, as Ravenstein (1885, particularly for the case of Britain) and Mabogunje (1970, particularly for the case of Africa) have already shown, migration is anything but a random phenomenon. In different geographical and historical settings, they both observed that most migrants move along spatially clustered pathways between very particular communities in origin and destination areas. Similarly, at a macro level, Zelinsky (1971), Skeldon (1990) and Hatton and Williamson (1998) observed clear long-term regularities between demographic, economic and social transitions on the one hand and the sequenced emergence and decline of particular forms of internal and international human mobility on the other.

## Paradigmatic classification of migration theories

Since the late-nineteenth century, various theories have emerged in various social-science disciplines which all aim at understanding the processes that drive migration. Such early migration theories can be clustered together into two main paradigms, following a more general division between ‘functionalist’ and ‘historical-structural’ social theory. Despite their various disciplinary origins, theories within each of these two main paradigms share basic assumptions about the nature of society and how society should be studied. For instance, neo-classical equilibrium models (from economics), push–pull models and migration systems theories (mainly from geography and demography) as well as dominant interpretations of migrant network theories (primarily from sociology) can all be situated within the *functionalist* paradigm of social theory, according to which migration is, by and large, an optimisation strategy of individuals or families making cost–benefit calculations.

Likewise, despite differences in nuance and level of analysis, neo-Marxist conflict theory, dependency theory (Frank 1966), world systems theory (Wallerstein 1974, 1980), dual labour-market theory (Piore 1979) and critical globalisation theory (see Sassen 1991) have broadly similar interpretations of migration as being shaped by structural economic and power inequalities, both within and between societies, as well as the ways in which migration plays a key role in reproducing and reinforcing such inequalities. All these theories can be situated within the *historical-structural* paradigm, also known as ‘conflict theory’, which focuses on how powerful elites oppress and exploit poor and vulnerable people, how capital seeks to recruit and exploit labour and how ideology and religion play a key role in justifying exploitation and injustice by making them appear as the normal and natural order of things.

More recent theories that focus on migrants’ everyday experiences, perceptions and identity – such as transnational (Vertovec 2009), diaspora (Cohen 1997; Safran 1991) and creolisation (Cohen 2007) theories – can all be situated within the *symbolic interactionist perspective* in social theory. We can perhaps distinguish a fourth, slightly more hybrid, group of meso-level theories that focus on the continuation or ‘internal dynamics’ (de Haas 2010b) of migration, such as network theories, migration systems theory (Mabogunje 1970) and cumulative causation theory (Massey 1990). We can thus reduce what may initially appear as a rather dizzying theoretical complexity by combining existing disciplinary theories (ranging from economics to anthropology) under the conceptual umbrellas of the main social-theory paradigms. For the sake of brevity and because the goal of this paper is to advance an understanding of migration processes as part of broader social change, the following analysis will focus on the classic distinction between functionalist and historical-structural theories.

## Limitations of functionalist and historical-structural theories

Functionalist social theory tends to see society as a system, a collection of interdependent parts (individuals, actors), somehow analogous to the functioning of an organism, in which an inherent tendency towards equilibrium exists. Functionalist migration theories generally see migration as a positive phenomenon contributing to productivity, prosperity and, eventually, greater equality in origin and destination societies through bidirectional flows of resources such as money, goods and knowledge. Essentially, they interpret migration as an optimisation strategy, in which individuals (and sometimes families or households) use migration to access higher and more-secure sources of income and other livelihood opportunities.

Neo-classical migration theory, as pioneered by Todaro (1969) and Harris and Todaro (1970), is the most prominent representative of functionalist migration theories but there are more theoretical currents that can be grouped under the functionalist migration paradigm. Push–pull models are basically a prototype version of neo-classical migration theories as they interpret migration as a function of income and other opportunity gaps between origin and destination areas. These functionalist models are all based on the explicit or implicit assumption that people make rational decisions in order to maximise income or ‘utility’. The only major exception on this rule seems to be the new economics of labour migration (NELM) pioneered by Stark (1978, 1991), which conceptualises migration occurring in contexts of relative poverty and constraints as a household’s or family’s (instead of an individual’s) co-insurance strategy aimed at diversifying (instead of maximising) income through risk-spreading. Although it acknowledges the role of structural constraints in shaping migration decisions, NELM is also ultimately based on the assumption that households are rational actors engaging in a long-term economic optimisation strategy.

At the macro level, from a functionalist perspective, individual optimisation decisions are expected to contribute to a more optimal allocation of factors of production – primarily through the transfer of labour from poor to rich areas and countries and concomitant reverse flows of capital from rich to poor areas – which is expected to decrease economic gaps between origin and destination areas (de Haas 2010a). However, such accounts typically ignore how poverty, inequality, immigration restrictions, government repression and violence can prevent people from migrating, cause their forced displacement or compel migrants into exploitative work conditions. This explains why the social and economic benefits of migration often accrue disproportionally to the already better-off in origin and destination societies – migrants and non-migrant ‘natives’ alike.

The central problem of functionalist migration theory is its reductionist character. The ‘push–pull’ reasoning on which these explanations are based strongly resonates with intuition but has proved to be inadequate and often plainly misleading in understanding real-world migration processes. Push–pull models are not able to explain migration *as a social process*, as they tend to list a number of static factors that obviously play ‘some’ role in migration but without specifying their role and interactions or providing a structural account of the *social processes* driving population movements. Skeldon (1990, pp. 125–126) therefore argued that push-pull models leave us with ‘a list of factors, all of which can clearly contribute to migration, but which lack a framework to bring them together in an explanatory system’, leading him to conclude that ‘the push–pull theory is but a platitude at best’.

If we reformulate the cornerstone functionalist assumption as ‘most people migrate in the expectation of finding better opportunities at the destination’, probably few would disagree. This assumption, that people basically have good reasons to move, however, is so general that it is of little use in explaining the geographically patterned and socially differentiated nature of migration processes. In other words: knowing what *motivates* individual people to move does not really help us to explain the processes, patterns and drivers of migration at the structural level.

The real questions are more complex and beg more complex answers. For instance, why do wealthier, more ‘developed’ societies tend to have *higher * levels of immigration and emigration than poor and ‘underdeveloped’ societies, while push–pull and neo-classical models would predict the contrary? How can we explain that most migration does not occur from the poorest to the richest societies? Why does ‘development’ in origin countries often lead to *increased* emigration propensities? And why do most people actually *not* migrate despite the existence of huge income and opportunity gaps within and between countries?

Functionalist migration theories have inherent difficulties explaining the socially and geographically differentiated nature of migration processes, in which structural inequality and discriminatory practices strongly favour the access of particular social groups and classes to attractive, legal migration opportunities, while excluding others by depriving them of rights or compelling them into exploitative situations. People’s ability to make independent migration choices is constrained by states and other structures such as family, community, networks and culture, which ultimately determine the social, economic and human resources which people are able and willing to deploy to migrate. At best, functionalist theories and push–pull models can incorporate such structural constraints as ‘market imperfections’ in theoretical models or cost-increasing factors in regression analyses. However, this exposes their inability to conceptualise how structural forces actively *shape* migration processes and often stand at the very origins of large-sale migration systems.

Colonialism, warfare, labour recruitment, migration policies, land dispossession, eviction due to infrastructure projects (e.g., road and dam construction) and cultural change are all examples of complex macro-structural change processes that cannot be reduced to ‘factors’ or ‘variables’ affecting migration costs. For instance, much large-scale migration has its origins in active efforts by states and employers to recruit foreign labour (see de Haas et al. 2020a; Piore 1979). This shows the need to conceptualise the role of states, businesses, recruiters and various other migration intermediaries (see Agunias 2009) as well organisations (such as the UNHCR, IOM or humanitarian organisations (see Olayo-Méndez 2018)) in *actively shaping migration processes* and creating entirely new migration patterns instead of reducing them to cost-increasing constraints in ‘naturally’ occurring migration processes. A full understanding of the role of such structural migration drivers therefore obviously defies their reduction to a few factors  in a mathematical theoretical model or variables in an empirical regression model.

Besides their general inability to conceptualise how structural factors and actors have actively shaped migration processes throughout history, functionalist theories have difficulties in explaining how, in the real world, migration can reinforce pre-existing inequalities. This upsets the underlying equilibrium assumption of functionalist theories, according to which social and market forces, if left to their own devices, would automatically tend towards equilibrium and in which (free) migration is thus expected to lead to income convergence and, eventually, less migration. Myrdal (1957) already argued that, without redistributive government intervention, socio-economic processes of ‘cumulative causation’ tend to reinforce inequalities between poor and rich areas, rather than the other way around. In the same vein, historical-structural theories argue that structures have, in fact, a tendency to reproduce or even reinforce inequalities, both ‘vertically’ between social groups (such as classes) and ‘horizontally’ across space (i.e., between peripheral rural areas and cities or between rich and poor countries).

For instance, in the poorest countries of Africa and Asia, legal migration opportunities to Europe, North America and other wealthy countries are mainly the prerogative of elite groups, who possess the right diplomas to qualify for a work or study visa and who have the financial means to pay for their migration. If it is mainly rather well-off groups who gain access to the most lucrative forms of legal migration, while others remain stuck in immobility or are relegated to exploitative forms of (often undocumented) migration, this is likely to sustain or even deepen inequalities within origin societies. As Van Hear (2014) argued, migration and its outcomes are “shaped by the resources that would-be migrants can muster and that in turn the capacity to mobilise such resources is largely determined by socio-economic background or class” (Van Hear 2014, p. 100).

Such arguments resonate with neo-Marxist political economy and historical-structural theories that emphasise how social, economic, cultural and political structures constrain and direct the behaviour of people in ways that do not generally create greater equilibrium but, rather, reinforce such inequalities. These theories emphasise the role of businesses – and states representing their interests – in shaping migration and see labour migrants, both forced and voluntary, as providing a cheap, exploitable labour force, mainly serving the interests of wealthy groups, areas and countries (de Haas et al. 2020a; Piore 1979). These theories emphasize that economic and political power is unequally distributed and that cultural beliefs (such as religion and tradition) as well as social practices serve to justify and reproduce such inequalities. From this perspective, it is thus the already privileged in destination societies, such as employers and capital-owning elite groups more in general, who mainly benefit from migration. This is reflected in migration rules and political discourses that favour and praise the skilled and wealthy and disdain or vilify the less-skilled, vulnerable, ethnically different and poor migrants and often relegate them to be exploited in the informal sector. From this perspective, the real aim of anti-immigration policies is not to stop immigration but to create an *appearance* of control (see Massey et al. 1998, p. 288), with the associated anti-immigrant political discourses serving to justify the economic exploitation of vulnerable migrant groups and to blame migrants for problems not of their own making.

However, the central problem of such historical-structural views is that they leave hardly any room for *human agency*. They tend to depict migrants as pawns – pushed and pulled around by global macro forces – or as victims of capitalism who have no choice but to migrate in order to survive. Views of migration as a ‘desperate flight from misery’, or that portray migrants as passive victims of smugglers and traffickers, do no justice to the fact that the vast majority of migrants move of their own free will. Indeed, a large body of research evidence shows that most migrants succeed in significantly improving their livelihoods through internal and international migration (de Haas et al. 2020a; Massey et al. 1998; UNDP 2009). In addition, juxtaposing mainstream narratives depicting smugglers as ‘unscrupulous and ruthless criminal gangs preying on vulnerable and desperate migrants’, the lived experiences of migrants expose a much more nuanced reality, with smugglers generally functioning as migration facilitators who can be close friends, acquaintances or more distant service deliverers (Zhang et al. 2018, p. 6).

Historical-structural views are often based on underlying assumptions that much 'South-North' migration is a largely irrational process that would  often not be in the interests of migrants themselves, as they would be blinded by over-optimistic mirages about life abroad and deceived by untrustworthy recruiters, smugglers and traffickers. This assumption is also reproduced in official discourses and policies according to which prospective migrants should be educated about the risks and costs of migration through information campaigns. This clearly denies the fact that, even for less-skilled or undocumented migrants, migration still has huge potential to improve the long-term wellbeing of themselves and their families and that they are therefore willing to endure situations of exploitation and suffering, however unjustified these may be from a moral and ethical point of view.

## Towards a more meaningful understanding of migratory agency

At first sight, functionalist and historical-structural accounts of migration seem diametrically opposed in their understanding of migration, in terms both of its social causes and of its consequences for destination and origin areas. However, what both paradigms have in common is a general inability to provide a meaningful understanding of human agency through their portrayal of migrants either as rather soulless individual utility-optimisers or as rather passive victims of global capitalist forces. Numerous studies have highlighted the limited but real ability of migrants to defy government restrictions, discrimination and xenophobia by migrating over closed borders, by buying in the services of recruiters, lawyers, smugglers and various other migration intermediaries, and by forging networks and new identities as well as establishing communities and their own economic structures in destination societies (Agunias 2009; De Haan et al. 2000; de Haas 2010b; Stark 1991; Zhang et al. 2018). It would therefore be just as unrealistic to depict migrants as victims desperately fleeing situations of destitution, opression and human misery as it would be to depict them as entirely rational and free actors who constantly make rational cost–benefit calculations. This shows that neither functionalist nor historical-structural theories provide realistic accounts of migratory agency. The central challenge in advancing migration theory is therefore the elaboration of conceptual tools that improve our ability to simultaneously account for structure and agency in understanding processes and experiences of migration, without discarding the important insights which both functionalist and historial-structural paradigms offer and thus rejecting them altogether.

## Theoretical exclusivism versus conceptual eclecticism

While the assumptions of neither functionalist nor historical-structural theories have universal value, both sets of explanations can nevertheless be useful in developing a richer, nuanced and contextualised understanding of migration processes. After all, functionalist theories may have greater explanatory value for some types of relatively unconstrainted forms of human mobility – such as much internal and high-skilled migration – while historical-structural theories may have greater explanatory value for forms of migration where government restrictions, exploitation and involuntariness play a more important role. Migration can be a very empowering experience but can, in other cases, take more exploitative forms.

Thus, instead of rejecting either set of explanations, insights from both paradigms need to be incorporated in a new, overarching theoretical paradigm on migration that can unite them. Instead of seeing theories as exclusive truth claims, we need a vision in which the validity of theoretical assumptions is contingent on the specific conditions under which migration occurs, the specific social and class groups concerned, as well as on levels of analysis. This implies that both the functionalist and historical-structural paradigms can have explanatory power and relevance and can, therefore, to a certain extent, be combined and integrated in a wider meta-theoretical framework which is able to simultaneously incorporate agency *and* structure in explaining migration and which acknowledges that the vast majority of migrants face some level of constraint yet also have some level of choice.

The way forward is therefore not to develop entirely new theories but to find concepts and analytical tools that help us to build upon and bridge insights provided by existing theories – not only within but also *across* paradigms. This implies a rejection of the somewhat common idea that we cannot combine social theories that are based on conflicting paradigmatic assumptions, particularly if we combine explanations at different levels of analysis.

A simple example may serve to illustrate the relevance of insights from both functionalist and historical-structural migration theories. From a macro-level perspective, some forms of migration seem rather exploitative – such as undocumented migration from Mexico to the US, Morocco to Spain, Mali to Côte d’Ivoire, Myanmar to Thailand or Indonesia to Malaysia – where uncertain legal status or ‘illegality’ enables employers to hire and fire migrant workers as they please and to pay low wages. At the macro level, such ‘exploitative’ forms of migration can exacerbate economic gaps between origin and destination areas by supplying cheap labour and boosting profits and income growth in destination areas for which reverse resource flows to origin areas such as remittances cannot compensate. Unequal terms of trade, higher productivity and economics of scale can lead to a further concentration of economic activities in wealthy destination countries along with the sustained migration of workers from poor countries to support them (see Martin and Taylor 1996). At the micro level, however, it may still make sense for people to migrate if this increases family income significantly and enables them to build a house, afford health care, send their children to school or start a small enterprise. The second insight does not prove the first wrong – vice versa.

This resonates with the argument of Massey and his colleagues (Massey et al. 1993, p. 432), who stated that there is considerable scope to combine insights from different theories:

A full understanding of contemporary migration processes will not be achieved by relying on the tools of one discipline alone or by focusing on a single level of analysis. Rather, their complex, multifaceted nature requires a sophisticated theory that incorporates a variety of perspectives, levels and assumptions.

Some migration researchers have countered this idea. For instance, Bakewell (2010, p. 1692) argued that Massey et al.’s (1993) claim that there are no necessary, inherent contradictions between different theories is hard to sustain ‘when one considers very different ontological and epistemological foundations of migration theories’. This is essentially is a Kuhnian argument on the incommensurability of scientific paradigms. As Kuhn (1962) argued in *The Structure of Scientific Revolutions*, the proponents of different paradigms live in different worlds and use different vocabularies and criteria determining the legitimacy of both problems and proposed solutions in terms of methodology and analysis. Each paradigm therefore has the tendency to satisfy the criteria it sets for itself and to reject the problem definition as well as evaluation criteria used by other paradigms (Kuhn 1962, p. 109). Importantly, such a Kuhnian view precludes combination or comparison across scientific paradigms, as adherents of different paradigms seem to be living in largely self-contained ‘truth bubbles’.

Kuhn’s view is extremely valuable for understanding the limited communication and the lack of recognition and appreciation among social scientists conducting research across paradigmatic, methodological and disciplinary divides. Yet there is also good reason to question the full applicability of the Kuhnian incommensurability principle to the social sciences (see also Urry 1973). Importantly, Kuhn based his argument on an analysis of the history of the natural sciences. In contrast to natural sciences, social theories typically have no universal bearing but are specific to particular historical and geographical contexts. The positivist universality claims of most natural-science theories can therefore not simply be extended to the social sciences, where theories need to be contextualised and historicised in order to make them meaningful and to be clearer about their specific applicability. Hence, in the social sciences, theories and paradigms do not need to be mutually exclusive a priori. Rather, they offer different explanations of social phenomena which can frequently be combined, particularly if they apply to different historical or social contexts, social groups or levels of analysis, or if they look at the same social phenomenon from different thematic, disciplinary and methodological angles.

It is therefore also dangerous to blindly apply the Popperian falsification principle to the analysis of the social world. For instance, if an empirical analysis conducted within a particular context shows that neo-classical ‘predictor variables’ such as wage differences do not have a significant effect on this particular form of migration, this still does not provide sufficient evidence to reject the theory as a whole. It may simply mean that a particular theory may have little or no explanatory power in that particular context. This does not provide a licence for a sloppy practice of ‘anything goes’ ad-hoc theorising but, rather, makes the case for *more analytical precision* in carefully assessing the applicability of particular theories in particular settings and at particular levels of analysis. Because theories have often been formulated to explain specific forms of migration occurring in particular geographical and historical contexts, a greater awareness of the history of theories is essential if we are to understand their particular claims and applicability.

Instead of ‘rejecting’ or ‘confirming’ hypotheses and theories, social analyses would gain interpretative depth and theoretical relevance if they indicate which contextual factors may explain certain expected or unexpected empirical observations. One single experiment defying Newton’s law of universal gravitation should indeed suffice to reject his theory. However, in contrast to the natural sciences, in social sciences it is often not about one theory being ‘right’ or ‘wrong’, let alone about testing the validity of an entire theory by plugging in a predictor variable ‘representing’ the theory in a regression model. In this context, Garip (2012, p. 425) rightly argued that migration researchers tend to ‘reduce theories to competing sets of independent variables ... [which] inevitably leads to either/or theoretical stances, rather than an emphasis on the complementarity of different theories’. Instead of stubbornly adhering to theoretical exclusivism, social theory-building should therefore be an inherently eclectic affair, in which an openness should exist to potentially combine different theoretical perspectives as part of an effort to develop more comprehensive, nuanced and, therefore, more realistic conceptual frameworks.

To summarise, migration theories can potentially be combined across five analytical dimensions:

- At different *levels of analysis*: macro-, meso- and micro-level explanations of migration may require different conceptual tools. For instance, forms of exploitative labour migration that seem to fit within the neo-Marxist paradigm can still be rational for migrants and their families.
- In different *(geographical, regional, national) contexts*. For instance, functionalist neo-classical theories may work better to explain relatively unconstrained migration in wealthier countries, while historical-structural approaches may be more useful to explain migration within and from poor or ‘developing’ countries or occurring under conditions of oppression and violence.
- Across *different social groups*: even at the same point in time and in the same geographical and national context, migration is a socially differentiated process; different theories are therefore likely to have varying degrees of applicability to different occupational, skill, income, class or ethnic groups. For instance, neo-classical assumptions may hold relatively well in explaining the migration of higher-skilled migrants, whereas neo-Marxist theories may be more useful in understanding the migration of less-skilled and relatively poor manual workers.
- At *different points of time*. The drivers and internal dynamics of migration processes often change over time and over the various trajectories and successive stages of migration system formation and decline (de Haas 2010b); so, too, therefore, do the social, cultural and economic mechanisms explaining such migration. For instance, Garip (2012) identified four distinct types of Mexico–US migrants over the 1970–2000 period and argued that these types gained prevalence during specific time periods depending on the changing conditions in the two countries.
- From *different thematic or disciplinary perspectives*. We can look at the same manifestation of migration from various analytical perspectives. For instance, we can study how social transformation processes shape migration processes simultaneously from cultural, political, economic, technological and demographic perspectives as well as through the use of various methodologies and data. This provides different – and generally complementary – angles from which to study and explain the same social process (see de Haas et al. 2020b)

This highlights the considerable potential to combine different theories to improve our understanding of migration processes across different levels of analysis (and aggregation), contexts, social groups and periods. In this way, disciplines, theories and paradigms become interpretative frameworks that reflect a particular way of viewing the world or dominating certain societies or periods. Such varying perspectives can be complementary (when they stress different dimensions of the same phenomenon) or may seem conflicting (when their fundamental assumptions clash) – although what initially appears to be a clash of assumptions may partly reflect their applicability to different contexts, social groups and levels of analyses. This shows the danger of buying into one particular train of thought, in which theories can easily devolve into intellectual straightjackets rather than conceptual toolboxes. This should compel us to achieve a deeper understanding of the concrete historical, geographical and social contexts in which migration occurs. It also highlights the need to break with bad habits of disciplinary and methodological parochialism.

## Migration as an intrinsic part of broader social change

A first essential step in our quest to achieve an more comprehensive theoretical understanding of migration is to connect migration theories to general social-scientific theories. This reflects the need to *(re) conceptualise migration as an intrinsic part of broader processes of economic, political, cultural, technological and demographic change* embodied in concepts such as social transformation, ‘development’ and globalisation.^1^ This is in opposition to more conventional scientific views which portray migration as either a response to development disequilibria or a function of static ‘push’ and ‘pull’ factors as well as policy views that portray migration either as a ‘problem to be solved’ or, conversely, as a solution to problems (such as population ageing). However, migration is a *social process* that cannot be seen in isolation from the broader processes of change of which it is a constituent part.

Urbanisation is perhaps the best example to illustrate this fundamental point. Since the onset of the industrial revolution, migration and urbanisation have been intrinsically intertwined processes which can therefore not be conceptualised separately. It is as unconceivable to understand modern urbanisation processes without understanding rural-to-urban migration as it is to understand rural-to-urban migration without understanding urbanisation processes. This exposes the flawed assumptions underlying attempts by governments to curb rural-to-urban migration through rural development programmes (see Rhoda 1983), as such policies cannot stop broader processes of social transformation and capitalist expansion which inevitably undermine traditional agrarian livelihoods, encourage the growth of the urban sector and irrevocably change ideas of the ‘good life’ amongst new generations towards more urban lifestyles (see Mabogunje 1970; Schewel 2019).

**Fig. 1:**
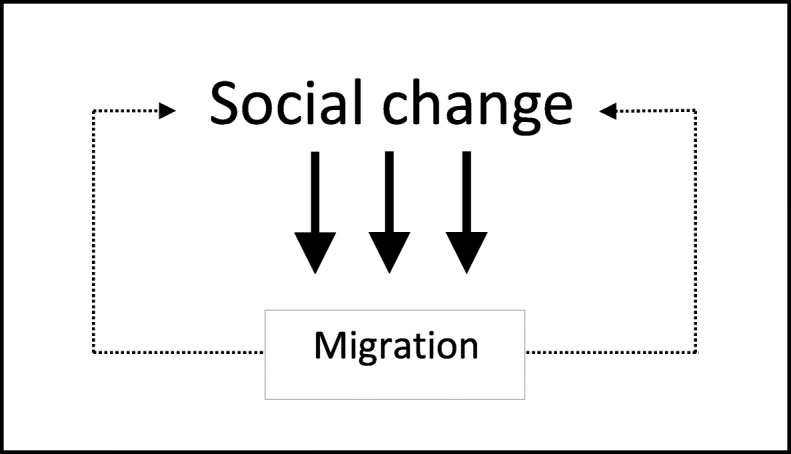
Migration as an intrinsic part of broader social change

While broader processes of social change shape migration, through its social, economic, cultural, demographic and political impacts, to some extent migration also affects these processes in its own right. For instance, remittances can increase income inequality and relative deprivation in origin communities and therefore further increase emigration aspirations, while large-scale immigration can affect the structure and segmentation of labour markets in destination countries (Massey 1990; de Haas et al. 2020a). Although this relationship is reciprocal, it also tends to be a highly asymmetrical one, because migration is generally unlikely to affect the deep structures of origin and destination societies unless it takes on truly massive proportions (see Portes 2010) or colonisers subjugate native populations through military force. This reciprocal but asymmetric relation between migration and broader social change is depicted in Fig. 1.

Prior research has shown that social transformation and economic development often shape migration in complex and often quite counter-intuitive ways that reveal the inadequacy of conventional migration theories. In low-income societies, marginal increases in income, improving education, infrastructure expansion, urbanisation and concomitant transformations from largely agrarian to industrial and service-based economic systems are generally associated with *increasing* levels of both internal and international migration. In other words, development initially leads to more migration. Only in the longer term, when societies become wealthier and predominantly urban, does emigration tend to decrease and immigration to increase, after which societies transition from being net emigration to net immigration countries. Such mobility or migration transitions were first hypothesised by Zelinsky (1971) and further elaborated on by Skeldon (1990, 1997). In 2010, I  elaborated a theoretical explanation of the social mechanisms underpinning the occurrence of migration transitions and, drawing on new data, I performed a first global test of the relation between levels of development and levels of immigration and emigration (de Haas 2010c). These empirical analyses confirmed migration transition theory and were further validated by subsequent studies (Clemens 2014, 2020; de Haas and Fransen 2018). Historical studies, such as of the European emigrations to the ‘New World’ between 1850 and 1914 (Hatton and Williamson 1998), also seem to confirm the transition model.

This non-linear relationship between development and migration levels clearly challenges functionalist, and historical-structural migration theories as well as push–pull models, which all implicitly or explicitly assume that the reduction of poverty and economic gaps will reduce migration. More in general, these insights highlight the need to conceptualize migration as a *normal* social process. As long as societies change (which they always do), social stratifications persist (which is equally likely) and people go through life stages (which is inevitable), people will keep on migrating – and settling. Societies are in constant mutation and migration should therefore be seen as a *normal* process, instead of being normatively cast as an undesirable or desirable process (in public debates), as the ‘antithesis’ of development (as in historical-structural accounts) or as a largely temporary response to development disequilibria (as in neo-classical accounts). The relevant theoretical question is therefore not ‘why people move’ (which tends to yield overly generic and rather meaningless platitudes of the ‘push–pull’ genre) but, rather, how patterns and experiences of migration are shaped by broader processes of social change.

## Conceptualising structure and agency in migration processes

The main conceptual problem of conventional theoretical accounts of migration remains their inability to meaningfully conceptualise how individual migrants and groups of migrants exert agency within broader structural constraints. Because of their centrality to our analysis and because of frequent confusion around their meaning, it is important to define the key terms of agency and structure. *Agency* reflects the limited – but real – ability of human beings (or social groups) to make independent choices and to impose these on the world and, hence, to alter the structures that shape and constrain people’s opportunities or freedoms. *Structure* can be defined as patterns of social relations, beliefs and behaviour. Factors and institutions such as class, religion, gender, ethnicity, networks and markets as well as cultural belief systems all sustain inequalities and social hierarchies and limit the opportunities that people have – or perceive they have – and the economic, social and cultural resources which they can access – thus significantly constraining their freedom or agency as well as their ideas, knowledge and self-consciousness.

As mentioned above, historical-structural theories tend to portray migrants as relatively passive actors or victims who are pushed around the globe by the macro-forces of global capitalism. Because they focus on the behaviour of actors (individual migrants) and may therefore come across as more ‘agentic’ at first sight, functionalist theories basically argue the same and do not ascribe much, if any, real agency – and therefore power – to migrants. Push–pull and neo-classical gravity models (the latter borrowed from the natural sciences) basically assume that people will migrate if the benefits of migration exceed the costs. This reflects an implicit assumption that people are motivated by individual cost-benefit calculations aimed at income or utility maximisation and will therefore react in automatic, universal and predictable ways to external stimuli or ‘push’ and ‘pull’ factors. As already embedded in the very semantics of the ‘push’ and ‘pull’ terminology, which we therefore need to reject, this reduces people to objects who operate in a social vacuum and who lack a will, perceptions and preferences of their own as well as the ability to actively and subjectively choose between different options.^2^ Functionalist theories and actor-focused empirical applications such as ‘agent-based modelling’, should, however insightful they can be, therefore not be misidentified as ‘agentic’ theories.

Conventional migration theories tend to ignore five vital issues with regard to migratory agency. First, people’s access to economic (material), social (other people), cultural (ideas, knowledge and skills) and bodily (good health, physical condition and habitus) resources shapes their ability to move (or, conversely, their ability to stay), their preferences and aspirations (to stay or to go), their choice in terms of destinations and their ability to obtain work, housing, education and legal status while protecting themselves against abuse and exploitation. Because of social hierarchies and structural inequality, such access to migratory resources tends to be unequally distributed within and across communities and societies.

Second, people’s perceptions of the ‘good life’ and, hence, their life aspirations, vary hugely across different social and cultural contexts. In addition, such aspirations are anything but fixed and tend to change as people move through their life course and as societies change. Depending on people’s subjective life aspirations as well as their (equally) subjective perceptions of opportunities ‘here’ and ‘there’, they may – or may not – develop a desire to migrate. It is therefore unrealistic to simply assume that dissimilar social groups will develop similar aspirations and tendencies to migrate even when exposed to similar set of external factors or stimuli or ‘push’ and ‘pull’ factors.

Third, people do not uniquely migrate out of an instrumental ‘means-to-an-end’ desire to achieve aspired levels of wealth or living standards but may also value migration for more intrinsic reasons – such as wanderlust, curiosity and an innate desire to break free and discover new horizons. This means that not only the ‘functional’ but also the intrinsic, subjective value which people ascribe to mobility should be given a serious place in migration theory. Across societies and throughout history, particularly young people have often harboured a strong desire to leave home – at least temporarily – for a variety of reasons, from the socio-psychological need to separate from their parents, proving their independence and as a *rite de passage* marking their transition to adulthood. ‘Gap years’ and working holidays are not necessarily a unique prerogative of privileged Western youth but can also be seen as a modern manifestation of a more universal intrinsic desire of many young people to move and discover the world – before settling down. Berriane et al. (2013), for instance, observed that quite a number of sub-Saharan migrants in Morocco frame their journey in terms of ‘adventure’ and the desire to try out life elsewhere and should not, therefore, be automatically crunched into the stereotypical categories of marginalised or ‘desperate’ economic migrants or refugees.

Fourth, conventional migration theories fail to incorporate mobility and immobility in the same conceptual framework. This is necessary because movement is as much the norm as is sedentary life – and many people experience both over their lifetime. Modern sedentary lifestyles assume residency – but changing residency requires migration. This implies that we need to embed our understanding of migration within broader theoretical frameworks that include non-migratory mobility and people’s desire to move as well as to stay put and have a place ‘to live’.

Last but not least, it is important to bridge the dichotomous divide between the study of voluntary and that of forced migration. While virtually all migrants face some level of constraint, ‘forced migrants’ also have some level of agency as, otherwise, they would not be able to move in the first place. Refugees exercise their agency as far as possible in even the face of appalling circumstances. It is only under extreme conditions such as slavery and deportation that agency may be discounted largely or completely (see de Haas 2009). Conversely, most migrants normally cast as ‘voluntary’ face considerable constraints. For instance, many migrants who primarily move for work do so because they face severe constraints on personal development at home and the range of migration options available to them tends to be limited by economic, political and social constraints.

The fact that all migrants face constraints challenges the conventional dichotomy between ‘forced’ and ‘voluntary’ migration. It appears, therefore, more appropriate to conceive of a continuum running from low to high constraints under which migration occurs, rather than applying a dichotomous classification of forced versus voluntary migration to much more complex realities in which all people deal with structural constraints, although to highly varying degrees. In this way, reductionist, dichotomous classifications between forced and voluntary migration (see also Richmond 1988) can be overcome or nuanced, in ways that enable us to integrate virtually all forms of migration into one overarching meta-conceptual framework.

Addressing these five challenges requires the elaboration of new concepts of human mobility that can simultaneously account for both agency and structure. However, this is easier said than done, and the crucial question is how to do this *in practice*. To achieve this, the following sections will argue how a meta-theoretical conceptualisation of migration as a function of *aspirations* and *capabilities* to move (1) expands the theoretical concept of human mobility to include movement and non-movement, (2) improves our ability to develop a richer and more realistic understanding of the ways in which macro-level change affects people’s migratory agency and (3) enables us to elaborate new, theory-derived migration and mobility categories.

## Migration as a function of capabilities and aspirations

In 2002, Jørgen Carling published a seminal paper  exploring the role of aspirations and ‘abilities’ in migration processes. He introduced the concept of ‘involuntary immobility’ to describe the phenomenon of the growing numbers of people living in Cape Verde (and poorer countries more generally) who wish, but do not have the ability, to migrate (Carling 2002). Analysing the case of wartime migration in Mozambique, Lubkemann (2008) also applied the concept of involuntary immobility to argue that the usual conflation of migration with displacement conceals a large category of people who suffer from ‘displacement in place’ through ‘involuntary immobilisation’ because warfare trapped them in the places they wanted to leave. While this concept has been mainly applied to origin societies, involuntary immobility can also be used to describe situations in places of destination when aspiring return migrants cannot go back because of a lack of resources, border controls or adverse conditions in origin countries. It may also describe situations in which migrants ‘in transit’ are immobilised if they become ‘stuck’ as a consequence of a lack of resources, violence, border controls or a combination thereof (see Collyer et al. 2012).

The systematic distinction between the ability (or capability) and the aspiration to migrate allows for richer, nuanced and more realistic migration categorisations. This also resonated with my own fieldwork in south-Moroccan oases between 1993 and 1994 (de Haas 1995, 1998) and between 1998 and 2000 (de Haas 2003, 2006), which inspired me to develop alternative ways of theorising migration, because conventional migration theories struck me as somewhat useless in explaining the migration dynamics I observed. Particularly during my fieldwork in the south-Moroccan Todgha valley (de Haas 2003, 2006), I was confronted with the following puzzle: despite significant increases in income and general living conditions over previous decades, out-migration from the Todgha valley to big cities in Morocco and, particularly, European countries like France, the Netherlands and Spain had continued unabated. This did not fit at all within neo-classical migration theories and push–pull models, which would have predicted decreasing emigration as a consequence of improved local living standards.

This inspired me to adopt the concepts of *aspirations* and *capabilities* as theoretical tools enabling me to better understand what I was observing (de Haas 2003, 2006, 2014b). I argued that, although local living conditions had improved significantly in preceding decades, people’s general life aspirations had increased faster, leading to growing migration aspirations. Improved education, increased media exposure alongside the regular return of the migrant ‘role models’ and exposure to their relative wealth had all contributed to rapidly increasing material and changing social aspirations of people living in the valley. Particularly international migration had become so strongly associated with material and social success that many youngsters had become virtually obsessed with leaving. This ‘culture of migration’ also contributed to rapidly changing ideas of the ‘good life’ and an increasing disaffection with traditional, agrarian lifestyles. So, growing aspirations and capabilities to migrate had inspired and enabled increasing numbers of people to leave the valley despite, or paradoxically rather *because of*, significant improvements in local living standards, income and education.

The core argument of this paper  is that the fragmented insights from different disciplinary theories can be integrated into a single meta-theoretical framework through conceptualising virtually all forms of migration as a *function of aspirations and capabilities to migrate within given sets of perceived geographical opportunity structures*, in which

- Migration *aspirations* are a function of people’s general life aspirations and perceived geographical opportunity structures.
- Migration *capabilities* are contingent on positive (‘freedom to’) and negative (‘freedom from’) liberties.

The concept of migration aspirations expands the notion of migratory agency into the subjective realm. This addresses the central shortcoming of functionalist and historical-structural theories, which implicitly assume that people respond to external ‘stimuli’ in quite uniform – and therefore predictable – ways. In this view, migration aspirations reflect people’s general life preferences as well as their subjective perceptions about opportunities and life elsewhere. Both general life and more specific migration aspirations are thus affected by culture, education, personal disposition, identification, information and the images to which people are exposed.

Aspirations are conceptually distinct, although empirically not independent from, capabilities. A good example is education in rural areas, which expands not only people’s skills and knowledge but also people’s awareness of alternative, consumerist, urban or foreign lifestyles. This often changes people’s notions of the ‘good life’ and they may subsequently start to aspire to migrate, *partly independently from ‘objective’ material conditions* at home. However, education may also increase aspirations in a different way, because it may prompt teenagers and young adults to start thinking that these new material and cultural lifestyles are actually within their reach – reflecting the notion of the ‘capacity to aspire’ (Appadurai 2004; see also Czaika and Vothknecht 2012). In this way, increasing capabilities can increase aspirations. Generally, preferences tend to change and material and consumerist aspirations tend to increase along with broader processes of social transformation usually associated with capitalist development and modernisation (see de Haas et al. 2020b). However, the extent to which changing preferences translate into migration aspirations depends on the degree to which people perceive that their subjective needs and desires can be fulfilled locally. However, in general, increased access to new ideas through education and the media tends to change people’s ideas about the ‘good life’ in such a way that it increases their desire to explore new horizons and move out of rural places towards towns and cities or foreign lands.

## The vital intrinsic dimension of migration aspirations

It is essential to distinguish the (1) *instrumental* and (2) *intrinsic* dimensions of migration aspirations. ‘Gap years’ and ‘lifestyle migration’ can be examples of the latter, while labour and student migration are examples of the former although, in practice, intrinsic and instrumental aspirations may occur simultaneously and often reinforce each other. Instrumental aspirations have received the most attention in research and are related to migration as a ‘functional’ or ‘utilitarian’ means to achieve another end, such as a higher income, higher social status, better health care, better education or, in the case of refugees, protection from persecution and violence. Intrinsic aspirations refer to the value which people may attach to the migration experience in and of itself, such as the joy and pleasure derived from exploring new societies, seeing the ‘bright lights’ of the city (Harris and Todaro 1970, p. 126), or experiencing the social prestige linked to proving oneself and enduring the suffering and taking the risks often associated with migration – to be subsequently seen as a ‘man (or woman) of the world’ or the social status, recognition and respect that usually comes with the ability to provide for the family.

People can also derive wellbeing from having *potential *access to mobility freedom, *irrespective of whether people use these freedoms or not*. The central idea is that the very awareness of having the freedom to move and migrate can add to people’s life satisfaction, in the same way that freedom of speech and religion, the right to organise protest marches or to run for office can contribute to people’s wellbeing, irrespective of whether or not they eventually use those freedoms. Conversely, if people do not enjoy such freedom, they are likely to experience this as a form of wellbeing-decreasing deprivation. For instance, many young Moroccans describe their country as a ‘prison’ because of European migration restrictions. In Carling’s (2002) terms, they feel stuck in involuntary immobility. This does not mean that they will all migrate if given the opportunity but the feeling of deprivation is real.

So, border walls or other migration restrictions might actually fuel the desire to get to the other side by creating an obsession with ‘getting out’ as soon as the opportunity presents itself, while full mobility rights might paradoxically decrease such aspirations. Before Spain introduced travel visas for Moroccans in 1991, it was common for young Moroccan adults to spend a few (summer) months or years in Spain, often with mixed motives of tourism, pleasure and work. The introduction of visa requirements largely cut off such free circulation, created a market for smuggling and increased the tendency to stay longer for those who still managed to get in – which encouraged the increasingly permanent settlement of Moroccans in Spain (de Haas 2014a). While numerous empirical studies have indicated that most people would prefer to stay home and many migrants wish to return to their countries of origin, the irony is that the very deprivation of mobility freedom or the expectation of the future tightening of migration regimes may actually encourage non-migrants to get moving (before it is too late) and for migrants to cancel return plans (out of fear of not being able to migrate again).

This is what Vezzoli (2015) observed in her comparison of migration patterns from Suriname, Guyana and French Guyana, three neighbouring countries located in South America. Guyana and Suriname became independent from Britain and the Netherlands in 1966 and 1975, respectively. French Guyana is a French *département* and, as a consequence, its inhabitants are full French citizens. The paradox is that more than half of the population of Guyana and Suriname live abroad despite – or paradoxically partly *because* – migration restrictions imposed by their former colonisers and other destination countries, as this has contributed to an obsession with ‘getting out’. In contrast, the French Guyanese tend to have a more relaxed attitude towards emigration because, as French citizens, they have full mobility rights. Apart from better social security and living conditions, this partly explains why emigration has remained at quite low levels (Vezzoli 2015).

Life aspirations can thus often include mobility freedom as an intrinsically valuable and wellbeing-enhancing right, experience and awareness. This intrinsic dimension of migration aspirations is not taken seriously in the predominantly ‘functionalist’ migration literature, or it is set apart as an entirely and essentially different category of migration – e.g., ‘lifestyle migration’ (Benson and O’Reilly 2009). We do know from empirical research, however, that intrinsic ‘adventure’ and ‘lifestyle’ motives are not the prerogative of privileged Europeans or North Americans but can also be common among other migrant groups, such as undocumented migrants in England (Bloch et al. 2011) or African migrants crossing the Sahara (see Berriane et al. 2013; Bredeloup 2008; Pian 2009). This highlights the need to put the intrinsic dimensions of mobility freedom centre stage when theorising migration.

## Migration as freedom

Although not developed to analyse migration, Amartya Sen’s (1999) capabilities approach, which he proposed to reconceptualise ‘development’, provides useful conceptual tools that *can also be fruitfully applied to analysing migration*, as it helps us to simultaneously grasp the instrumental and intrinsic dimensions of migration capabilities and aspirations as well as to conceptualise how migration is an intrinsic part of broader development and change. Based on his critique of narrow, income-focused definitions of development, Sen (1999) conceptualised development as the process of expanding the substantive freedoms that people enjoy. He operationalised this through the concept of *human capability*, which he defined as the ability of human beings to lead lives they have reason to value and to enhance the substantive choices they have. Sen  argued that income growth itself should not be the litmus test for development theorists but the question whether the capabilities (or freedoms) of people to control their own lives have expanded. Sen posited that freedom is central to the process of development for two reasons. First, there is the *intrinsic* importance of human freedoms in directly adding to people’s quality of life, which has to be distinguished from the second, *instrumental*, value of freedoms in also contributing to human and economic progress (Sen 1999).

I initially applied Sen’s capabilities approach to the study of migration to evaluate the development impacts of migration and remittances in origin communities, not only in terms of income, but also in terms of wellbeing-enhancing improvements in living standards. Yet I disovered that the capabilities approach was also a valuable concept to understand how, conversely, processes of social transformation and development shape migration (de Haas 2003, 2006, 2010a). Changes in economic, social, cultural and political conditions in origin areas may affect migration propensities in two different ways. First, economic growth and improvements in living standards are likely to increase people’s migration capabilities by increasing their ability to assume the costs and risks of migrating. Second, the extent to which local opportunities allow people to lead the lives they have reason to value (which reflects Sen’s definition of development) at home is also likely to affect their migration aspirations.

Thus, by applying Sen’s capabilities approach to migration, we can learn to see migration not only as an instrumental-functional means-to-an-end to improve people’s living conditions but also as a potentially wellbeing-enhancing factor *in its own right.* This alludes to the intrinsic, wellbeing-enhancing (non-instrumental and non-utilitarian) dimension of migration and this, at a philosophical level, also expands our understanding of mobility not so much as the act or capability of moving but as the *ability to decide where to live, including the option to stay at home*. Based on this definition, people may enjoy mobility freedoms without ever using them, while migration can only be seen as genuinely wellbeing-enhancing and empowering if people also have the option to stay. This distinction between the *intrinsic* and the *instrumental* dimensions of migration enables us to go beyond common functionalist, instrumentalist views on migration, in which:

- the *intrinsic dimension of migration* is the direct contribution of the freedom of mobility to people’s wellbeing, irrespective of whether they move or not (‘migration as freedom’). It relates to (particularly young) people’s innate desire for adventure, discovery and separation from (the parental) home for shorter or longer periods as well as to the intrinsic wellbeing derived from the awareness of having the optional freedom to move. Such freedoms do not have to result in actual movement in order to be enjoyed: it is the very awareness of having the option – or freedom – of staying or going where one wants that matters most;
- the *instrumental (functional, means-to-an-end) dimension of migration* reflects the role of migration as a way to achieve other personal or family goals such as increased income, education, living standards or, in the case of refugees, personal safety. While people need a certain level of capabilities to be able to migrate, migration can further increase such capabilities. This corroborates the idea that migration is often quite literally an investment of families and individuals in a better future rather than a ‘desperate flight from misery’ as dominant discourses on migration often tend to frame it.

Usually, migratory agency is associated with the act of moving and setting up residency in another place or country. This reflects, however, a one-sided view since, after all, real agency also involve the option to *not* act (see Emirbayer and Mische 1998), as long as a real choice is present. A truly agentic view on migration should therefore capture both non-migratory and migratory behaviour. There is a long-standing controversy in the migration literature about whether migration or sedentary behaviour is the norm. The first argument is that migration is a universal part of the human experience and that we tend to erroneously misrepresent past societies as largely ‘immobile’, with migration being the ‘normal’ pattern. The second argument is that most people, if given the choice, prefer to stay at home (the ‘home preference’) and that migration is in fact rather limited in magnitude if we consider the huge economic inequalities across the globe.

Yet from a theoretical point of view this debate seems somehow futile. First, a truly agentic view on migration does not presume either moving or moving as the norm but, rather, acknowledges that they are two sides of the same freedom-of-mobility coin. Second, at both the practical and the conceptual level, migration is only a meaningful and relevant category in the context of sedentary lifestyles (as the concept of migration implies a change in residence). The lifestyles and livelihoods of hunter-gatherers and nomads are often characterised by permanent mobility and a lack of permanent residence – which renders a category like migration rather obsolete and meaningless. So, migration presumes sedentarism as much as sedentarism presumes migration. The residential lifestyles of both agrarian and capitalist-industrialised societies have thus ‘created’ the need for migration as a continuous adaptive response to social change and social transformation as well as a linguistic and conceptual category.

Third, considering that people make migration decisions as members of social groups, migratory and sedentary behaviour are often interrelated. For instance, one of the strengths of new economics of labour migration (Stark 1978, 1991) is the idea that migration is a strategy by rural households to diversify their income portfolio through the migration of one or a few family members. This means that there is often a strong co-dependency between non-migrant and migrant family members. This implies that migration matters to most of us, whether we move or not. Although only about 3% of the world’s population has migrated across borders and a roughly estimated 12% within borders (de Haas et al. 2020a), most people in the world are affected by migration in direct or indirect ways, either through family and other social ties or through the impacts of migration on origin and destination societies more in general.

## Redefining human mobility

Migration and sedentary behaviour are thus interconnected. We therefore need a truly agentic understanding of human mobility that can simultaneously capture movement and non-movement. Following Sen’s general argument on the intrinsic wellbeing-enhancing value of human freedoms, we should therefore also conceptualise the very capability to move (migrate) as a fundamental human freedom. To capture the idea of migration as a freedom in its own right, we should define *human mobility* not by the criterion of actual movement but as *people’s capability (freedom) to choose where to live* – with migration as the associated functioning (see also de Haas 2009; de Haas and Rodríguez 2010). Essentially, human mobility thus includes the freedom to stay, which we can classify as *voluntary immobility* (contrasting Carling’s (2002) concept of *involuntary immobility*).

This is related to the concept of capabilities in two different ways: first, people need access to social (other people), cultural (ideas, knowledge and skills^3^) and economic (material) resources if they are to exert migratory agency. Under highly constrained conditions of poverty and oppression, people often lack the resources to leave. Second, if people have no realistic option to remain – for instance through war, persecution, deportation or eviction by governments – or if they are pressured by their families to work abroad, they may feel deprived of an essential part of their human mobility freedoms, which is the option to stay. Conversely, if people feel deprived of the capability to move, the concomitant frustration of being ‘trapped’ may fuel migration aspirations and can even create an obsession with ‘getting out’.

Drawing on the capabilities–aspirations framework, Table 1 elaborates a theoretical categorisation of five ideal-typical individual mobility types. Based on our new definition of mobility as ‘people’s capability (freedom) to choose where to live, including the option to stay’, this categorisation of mobility types also includes various forms of *immobility*. This enables the theoretically desirable inclusion of both movement and non-movement within the same conceptual ambit as manifestations of the two sides of the same freedom-of-mobility coin.

**Table 1:** Aspirations–capabilities-derived individual mobility types

		Migration capabilities	
		*** Low***	*** High***
**Migration aspirations**(intrinsic and/or instrumental)	***High***	**Involuntary immobility**^a^ (feeling ‘trapped’)	**Voluntary mobility** (most forms of migration)
	***Low***	**Acquiescent immobility**^b^	**Voluntary immobility** *and* **involuntary mobility** (e.g. refugees, ‘soft deportation’)^c^

^a^(Carling 2002); ^b^ Schewel (2015, 2020); ^c^ See Boersema et al. (2014)

This categorisation builds upon Carling’s (2002) ‘involuntary immobility’ concept but expands with four other mobility types. It acknowledges a reality in which (cultural) preferences, aspirations and capabilities are deeply affected by macro-structural factors. It is only possible to speak about the ‘voluntariness’ of mobility or immobility if there was a reasonable option to stay. That does not mean that refugees and other groups of ‘distress migrants’ do not have any agency (otherwise they could not have moved in the first place), but that their migration is forced to the extent that they have been deprived of mobility freedoms; they had no real option to remain as that would have put them in serious danger of being persecuted, injured or murdered. For refugees, migration is primarily a response to severe danger at home rather than a positive response to opportunities elsewhere. Obviously, once a decision to leave has been made, such opportunities will play a role in deciding how, when and where to go and people will try to exert their agency as much as possible, although they cannot be conceptualised as the main reason to migrate. Thus, refugees are forced migrants because they had no option to remain.

Similarly, migrants who may be classified as ‘voluntary return migrants’ by governments or international organisations may only be ‘willing’ to return not out of a real, intrinsic desire to do so but because they either have no access to social amenities and shelter or they risk imprisonment, violence and other abuse (such as separation from their children) in destination countries. Under such situations of extreme distress and pressure, migrants may eventually decide to return and be compelled to sign forms confirming consent to their ‘voluntary repatriation’, even if this is against their own intrinsic preferences or desires (see Cleton and Chauvin 2020). Boersema et al. (2014) referred to this category as ‘soft deportation’. From a theoretical perspective, we can classify this as ‘involuntary mobility’. Even if such migrants are not literally forced to move (i.e., by violent means through deportation) they may, under severe threat, feel compelled to move even if is this strongly against their own intrinsic desire. The categories ‘voluntary mobility’ and ‘voluntary immobility’ only apply to people who have the capability to migrate but also have a reasonable option to stay (with the term ‘reasonable’ implying that this would not put them in dangerous, highly exploitative or life-threatening situations) and for whom the decision as to whether or not to go is primarily affected by their (instrumental or intrinsic) migration aspirations.

A final category concerns people with low capabilities *and* aspirations to move. How can we categorise a person living in poverty, who is neither able to migrate nor has ever imagined doing so? Based on the idea that capabilities affect aspirations (Appadurai 2004, see also Czaika and Vothknecht 2012), we may perhaps say that this person is deprived of the capability to aspire as well as the capability to move. This raises the philosophical question as to what extent we can call this form of immobility “voluntary”? Schewel (2015, 2020) therefore proposed the category of *acquiescent immobility* to describe situations in which people are neither able to migrate nor desire to do so. Schewel argued that because ‘acquiescent’ implies an acceptance of constraints (the Latin origins of the word meaning ‘to remain at rest’) this may be an appropriate term to describe this mobility category. However, we clearly need more research on the formation of aspirations to move or to stay and on the extent to which decisions to stay can indeed be seen as ‘acquiescent’ or, rather, reflect a *post-hoc* rationalisation of mobility deprivation.

## Positive and negative liberty as manifestations of structural conditions

As argued thus far, the capabilities approach enables us to conceptualise human mobility (people’s freedom to choose where to live) as a wellbeing-enhancing capability in its own right (‘migration as freedom’). In order to further enhance our understanding of how these individual migration capabilities and aspirations are shaped by, and interact with, macro-structural processes, it is useful to draw on the distinction between positive and negative liberties made by Isaiah Berlin and to apply it to the study of migration. In his *Four Essays on Liberty*, Berlin (1969) made a fundamental distinction between negative and positive liberty (or freedom). In brief, the concept of *negative liberty* refers to the absence of obstacles, barriers or constraints. This comes close to popular ways of conceiving freedom, which often focus on the role of governments in imposing constraints on people’s freedom or even being an outright threat to people’s lives, for instance through regulation, oppression, violence or war. *Positive liberty* refers to the ability to take control of one’s life and to realise one’s fundamental purposes. As Berlin argued, positive liberty ‘derives from the wish on the part of the individual to be his own master’ (Berlin 1969, p. 131). While Berlin’s argument focused on people’s role in choosing who governs society, this concept is also applicable to the ability – or agency – of people to actively change their life circumstances. Berlin’s concept of positive liberty therefore comes very close to Sen’s concept of capabilities as people’s ability to enhance the substantive choices they have.

The twin concepts of negative and positive liberties provide a useful conceptual link between the macro-structural conditions which shape these liberties on the one hand and people’s individual aspirations and capabilities – concepts which embody choice and agency but which are ultimately constrained by these structural conditions – on the other. From this perspective, the absence of external constraint (negative liberty) is not a sufficient condition for people to exert migratory agency, because they need a certain degree of ‘positive liberty’ that will enable them to enjoy genuine mobility freedom – which implies a real choice about where to live. For instance, governments may grant nominal freedom of movement but poor people may still lack positive liberty in the form of capabilities and access to resources that would enable them to actually use such negative liberty.

People may aspire to flee situations of poverty, distress and danger but they still need certain ‘positive liberties’ (capabilities) in the form of resources such as money, social connections, knowledge and physical ability, in order to be able to flee. The most vulnerable populations may not, therefore, have the option to flee and may be trapped into ‘involuntary immobility’. Poor people often only migrate if forced by conflict or disasters – and then mainly move over short distances – while the most vulnerable are often deprived of the possibility to move at all. For instance, when Hurricane Katrina hit New Orleans in 2005, many of the (car-less) poor were trapped in the city (Gemenne 2010; see de Haas et al. 2020a, p. 37). In the civil conflict that broke out in Libya in 2011, hundreds of thousands of guestworkers from sub-Saharan Africa were trapped in the country and exposed to abuse, violence, imprisonment and sometimes murder. In contrast to high-skilled migrants from European or other powerful states, they were immobilised because they lacked the resources and connections to move out (de Haas and Sigona 2012). This insight has important consequences for the way we analyse migration. For instance, scenarios that predict massive international migration as a result of climate-change are rather unrealistic partly because they ignore evidence that the most vulnerable populations who are most at risk to be negativelly affected by climate change generally lack the resource to move over large distances. In fact, deprivation as a result of climate change-driven environmental change (such as an increased incidence of droughts or flooding) may  deprive them from the capabilties to go elsewhere and therefore immobilize them in situ (see Foresight 2011; de Haas 2020).

This perspective helps us to understand the complex, often non-linear and frequently counter-intuitive ways in which macro-structural processes of social transformation shape trends and patterns of migration, because negative and positive liberties often impinge in quite different ways – and sometimes opposite directions – upon migration aspirations and capabilities. This renders the analysis of the effects of macro-structural conditions on migration patterns far from straightforward: although the deprivation of negative and positive liberties and awareness of better opportunities elsewhere may increase people’s migration aspirations, the absolute deprivation of the same negative or positive liberties, or both, may prevent people from exerting migratory agency. Conversely, while increases in negative and positive liberties may increase people’s mobility freedom, this does not necessarily lead to more migration as, under such conditions, more people may also be able to realise their intrinsic preference to stay through an increased ability to meet their life aspirations at home. Likewise, as we have seen, increasing mobility freedoms through the liberalisation of migration regimes may paradoxically decrease long-term, permanent emigration as it may take away people’s obsession with ‘getting out’.

## The structural formation of migration aspirations and capabilities

**Fig. 2:**
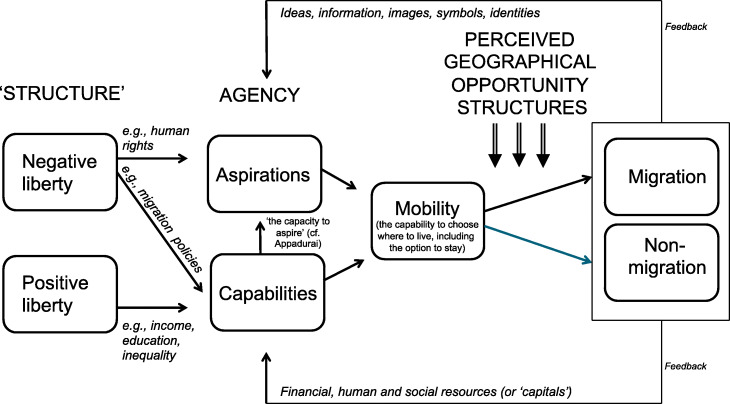
Expanded aspirations–capabilities framework for conceptualising migratory agency

Figure 2 depicts the various ways in which life aspirations and capabilities are affected by structurally determined positive and negative liberties and how these may affect mobility freedoms and people’s migration decisions. Negative liberty affects both people’s life aspirations and capabilities; the interaction between these factors explains complex, sometimes counter-intuitive migration outcomes. For instance, while it may seem likely that political oppression and violence will increase migration aspirations, the same factors may also deprive people of the capability of moving – such as through exit restrictions – or actually prompt them to stay so that they can protect family and community members. The concepts of negative and positive freedom therefore enable the incorporation of the role of states and policies in migration theories. From this perspective, mobility deprivation can happen either through negative liberty deprivation – for instance when authoritarian states deprive their citizens of the right to leave – or through positive liberty deprivation – when people lack the access to social, cultural and economic resources needed for realising migration aspirations.

Positive liberty primarily affects people’s capabilities in the form of their access to social, economic and cultural resources or ‘capitals’. Indirectly – and drawing on the notion of the ‘capacity to aspire’ (Appadurai 2004; Czaika and Vothknecht 2012) – increased capabilities are also likely to increase aspirations, by (1) making people aware of alternative opportunities and lifestyles and by (2) making people believe that migration is ‘within their reach’, that they can actually ‘make it’. For instance, acquiring a school or university degree is likely not only to increase knowledge about opportunities elsewhere but also to instil the belief and self-confidence that it is actually possible to find a job, to live in a strange place or to secure a visa.

Embracing a more ‘modern’ lifestyle, with its concomitant increase in perceived material needs, only prompts aspirations to migrate if people believe that their life goals cannot be fulfilled locally within the foreseeable future *and* if they believe that better opportunities exist elsewhere. Elaborating on Hirschman’s (1978) ‘exit or voice’ hypothesis we can argue that, if people are discontent, they can either try to change these circumstances (by raising their voice), consent (acquiesce) or leave. Thus, the imagined opportunities for future local change and people’s belief in their own power and moral obligation to contribute to such change will affect the extent to which increased life aspirations will translate into migration aspirations. A mismatch between personal life aspirations and conditions at home do not therefore necessarily translate into migration: they can also be fulfilled at home – for instance by starting up a business, pursuing an education, joining political movements or taking up arms. It only translates in migration aspirations if people lack, or lose, belief in future local change.

In order to simultaneously incorporate structure and agency migration theory, we need to connect both concepts and conceptualise their dialectics. In this respect, ‘structure’ is often unilaterally seen as a set of constraints. However, this is too limited a view. Essentially, structures are about *patterns* – or regularities and repetitions in social relations and social behaviour. Structures thus simultaneously constrain the migration of particular social groups while facilitating the migration of other groups along very specific geographical and social pathways. For instance, states and their policies have a strong structuring effect on migration, which means that they can facilitate and actively stimulate (particularly through recruitment) the movement of some (national, age, gender, skill, class and ethnic) groups while simultaneously hindering the movement of others.

Government policies, recruitment and other macro-structural factors thus shape socially differentiated, highly specialised and geographically bundled pathways (also known as ‘migration corridors’) linking very particular social groups and places over space. Once such initial patterns are set, migrant networks, feedback processes known as ‘cumulative causation’ (Massey 1990) and various other ‘internal dynamics’ (de Haas 2010b) tend to give migration processes their own momentum and thereby reproduce such patterns, giving migration an “identifiable geographical structure that persists across space and time” (Mabogunje 1970, p. 12). However, notwithstanding the importance of such internal dynamics in perpetuating migration and the consolidation of migration systems, the crucial point is that governments, employers and recruiters often play a key role in setting *initial* migration patterns that are subsequently reproduced over space and time.

The entire set of structural conditions at home and in imagined migration destinations creates complex opportunity structures, endowing different individuals and social groups with various sets of negative and positive liberties, which, depending on how these structural conditions affect people’s capabilities and aspirations and how people perceive these conditions through their social, cultural and personal lenses, may, or may not, make them decide to migrate. In turn, such migratory agency reciprocally affects these structural conditions through various feedback effects, which may stimulate more migration over the paths beaten by initial (pioneer) migrants (de Haas 2010b).

The aspirations–capabilities framework therefore enables us to explain why social transformation or ‘development’ is initially associated with increasing migration levels (see Skeldon 1990, 1997, 2012; de Haas 2007, 2010c). Particularly if regions or countries transform from a low-income, agrarian and peripheral status to a middle-income, industrialising and urbanising status, migration aspirations and capabilities both tend to increase rapidly, explaining the paradox of development-driven emigration booms. As long as people’s ideas of the ‘good life’ (generally away from traditional rural-agrarian lifestyles^4^) and the associated growth in material aspirations change faster than and outmatch local opportunities, this typically leads to *growing* migration propensities.

Only in the longer term, when local opportunities start to increasingly match aspirations, can we expect migration propensities to go down. While such capabilities and aspirations manifest themselves at an individual level, they are ultimately shaped by macro-structural changes such as the expansion of infrastructure, education and the media. This exposes the potential of the aspirations–capabilities approach to link macro-structural change processes to individual perceptions, experiences and agency with regards to migration decision-making.

## Towards new migration categories

As manifestations of structural conditions, the concepts of positive and negative liberty are also useful in operationalising ‘structural conditions’. This enables the development of a four-pronged typology of migration categories as presented in Table 2, which represents an ideal-typical categorisation of concrete manifestations of migration under different contextual configurations of relatively high and low positive and negative liberty. Table 2 also indicates the explanatory relevance of some of the main migration theories for these different contextual migration categories.

**Table 2:** Theoretical migration categories based on positive and negative liberty types

		Positive liberty (‘freedom to’; capabilities)	
		Low	High
**Negative liberty** (‘freedom from’; external constraints)	Low	**Precarious migration** Generally short-distance, often internal, by relatively poor or impoverished people vulnerable to exploitation, i.e., poor rural-to-urban migrants, undocumented labour migrants, ‘failed’ asylum-seekers, internal displacees) (relevant theories: historical structural; dual labour-market)	**Distress migration** Deprivation of mobility freedom through absence of reasonable option to stay; applies to refugees fleeing potentially life-threatening conditions but possessing the resources to move abroad and obtain legal status (relevant theories: historical structural; network; new economics of labour migration)
	High	**Improvement migration** Internal and international, often through networks, recruitment and pooling of family resources (relevant theories: new economics of labour migration; network and internal dynamics; cumulative causation; dual labour-market; mobility transition)	**‘Free migration’** relatively unconstrained mobility in and between wealthy countries or by wealthy people, skilled workers, ‘lifestyle’ migrants (relevant theories: neo-classical; human capital; mobility transition)

The categorisation presented in Table 2 is tentative and would benefit from further elaboration, verification and refinement. This main purpose of this effort is not to propose a ‘definitive’ categorisation of migration but, rather, to illustrate how the meta-theoretical framework presented in this paper can be helpful in developing a more systematic way of ‘contextualising’ the assumptions of the different theories and, in so doing, achieving greater precision when specifying the varying applicability of different theories. This exemplifies my position that theoretical assumptions should be seen as contextualised statements (or generalisations) rather than mutually exclusive truth claims.

As mentioned earlier, ‘neo-classical’ theories have a relatively higher relevance to explain the more or less *free migration* of relatively well-off people, under relatively unconstrained conditions characterised by high levels of positive and negative liberty. In their turn, neo-Marxist and other historical-structural theories may be comparatively more powerful in understanding and interpreting the *precarious migration* which takes place under highly constrained conditions – such as migration restrictions or the lack of state protection against abuse and discrimination. Such constraints reduce the agency of migrants, making them more vulnerable to exploitation by employers, recruiters, state agents or smugglers – and often frustrate their attempts to achieve upward socio-economic mobility through study and work. This can apply to both impoverished labour migrants and the majority of people forcibly displaced by conflict, disasters or persecution who lack the means and contacts to move over large distances. Although they had the resources and the ‘positive liberty’ to leave, they are at high risk of becoming trapped or ‘involuntarily immobilized’ along the journey or at the destination, deprived of the resources and freedom to continue the journey or to return.

In the category of *improvement migration*, people have relatively low levels of positive liberty (such as manifested by limited financial resources) but face relatively high negative liberty (such as manifested by access to legal migration opportunities and residency in wealthier countries) – which creates the conditions under which migration can be a successful way of achieving upward socio-economic mobility. For instance, recruitment programmes have historically given relatively poor people access to work opportunities abroad, thus fitting within dual labour-market theory (Piore 1979). Under other circumstances, family members often pool their resources to invest in the migration of one or more family members. This seems to fit with the assumptions of the new economics of labour migration (NELM), which conceptualises migration as a risk-sharing strategy by households aiming to diversify their income, generate remittances and improve the long-term wellbeing of the family (Stark 1978, 1991). Theories on the internal dynamics of migration processes (Massey 1990; de Haas 2010b) help to explain how migration often facilitates further migration through cost- and risk-lowering network effects. This explains the partial self-perpetuation of such migration even after recruitment has stopped and the original causes of migration such as labour demand have fallen away. This set of theoretical explanations seems to apply, for instance, to Mediterranean ‘guestworkers’ who were initially recruited to work in North-West European countries in the post-WWII decades as well as to Mexican migrant workers who were recruited to work in the US through Bracero programme between 1942 to 1964.

In other situations, people may face high levels of external constraint (negative liberty, such as through oppression, persecution or violent conflict) but still manage to migrate through their access to financial, social and human resources (positive liberty). Examples of this category could include skilled and/or relatively well-off refugees who are actually able to make it to other countries and obtain legal residency. This category, which I have tentatively named *distress migration* is a form of forced migration that needs further elaboration because existing migration theories seem to apply less easily to this category – reflecting the low level of theorisation of refugee migration. However, based on our definition of human mobility as the capacity to choose where to live, refugees are thus forced migrants *because they have no reasonable option to stay* even though they have some degree of agency in terms of having the resources and capacity to escape, move to another country and choose where to go.

There seem to be two basic ways of conceptualising ‘forced mobility’ – either as a conscious act to escape external threats and oppression (negative liberty deprivation) or livelihood insecurity and poverty (positive liberty deprivation) or as a literally forced mobility – such as through eviction, deportation or enslavement. In the first case, migrants still have agency and migration can be instrumentally or intrinsically voluntary; in the latter case, agency is entirely ruled out. For distress migrants, the existence of certain positive liberties – for instance speaking the host country language, having family already residing in the country, having the means to pay for a journey that is farther from home – can further increase both their aspiration and capabilities to migrate.^5^ While ‘distress migrants’ have some level of positive liberty and agency (or capability) over where they choose to go, ‘precarious migrants’ lack such resources, generally move over short distances, are more likely to get stuck in situations of ‘involuntary immobility’ and are more vulnerable to exploitation and extortion by state agents, employers or smugglers.

While Table 2 gives an indication of which migration theories seem the most relevant and the ‘best fit’ for these migration categories, it does not mean that each theory exclusively applies to that category but, rather, to where they seem to have the strongest explanatory power. As I argued earlier, there is considerable leeway to combine theories, particularly when applied to different levels of analysis. As argued above, although much migration of less-skilled workers may appear to be exploitative from a macro-structural point of view (fitting historical-structural theory), it may often still be beneficial from the point of view of individual migrants and their families (fitting neo-classical or NELM theories). Migrants may also shift categories over time – for instance if a restrictive turn in policies or increasing racism turns ‘improvement migration’ into ‘precarious migration’, while the reverse may, for instance, apply if ‘precarious migrants’ get access to legal status through regularisation campaigns. The applicability of these categorisations also depends on the type of migration. For instance, the same person may have the positive and negative liberties enabling her to migrate internally but lack the resources and access to documents enabling her to migrate across borders.

## Conclusion

This paper has elaborated an aspirations–capabilities framework to advance a new, comprehensive understanding of human mobility as an intrinsic part of broader social transformation processes. Drawing on previous work (Carling 2001, 2002; de Haas 2003, 2009) on aspirations and capabilities, this paper has expanded these concepts and embedded them in the wider theoretical perspectives on capabilities in development theory offered by Sen (1999) as well as the distinction between negative and positive liberty posited by Berlin (1969). While neither Sen nor Berlin developed these concepts to explain migration, this paper has argued that they can be fruitfully applied to migration to provide a richer, more agentic and realistic understanding of migration processes. Arguing in favour of conceptual eclecticism to bridge disciplinary and paradigmatic divides, this paper has shown the conceptual exigency and theoretical benefits of conceiving migration as an intrinsic part of broader processes of social transformation. Such a conceptualisation requires the embedding of the analysis of migration into general theories of societal change without reverting to the top-down causal determinism of conventional (historical-structural or functionalist) migration theories.

Except for extreme situations like slavery and deportation, migrants are neither passive subjects nor actors who react in automated and uniform ways to sets of ‘push’ and ‘pull’ factors – whether these be the macro-forces of global capitalism, wage gaps, violence or environmental stress. In order to migrate, people need to take the active decision to move and have the resources to do so. While historical-structural theories tend to portray migrants as passive pawns or victims of the forces of global capitalism, neo-classical and other functionalist migration theories implicitly assume that people’s preferences and, hence, life aspirations are constant across societies and over time, mostly boiling down to individual income (or ‘utility’) maximisation. This reveals that functionalist migration theories, despite their guise as ‘actor-focused’ models, are socially sterile and devoid of any real sense of agency, as individual choices are supposed to be entirely predictable outcomes of individual cost–benefit analyses based on fixed, static sets of assumed preferences.

The crucial flaw in this type of thinking is the assumption that people’s perceptions and preferences are (1) driven by individual utility maximisation; that (2) people’s preferences are uniform across societies and that (3) such preferences are static. So, in various ways, conventional migration theories all tend conceptualise migrants as persons being ‘pulled’ and ‘pushed’ like atoms by somewhat abstract economic, political, demographic or environmental causal forces. This ignores the fact that factors such as culture, education and exposure to media and other sources of images, ideas and knowledge are likely to have a huge impact on (1) people’s preferences and notions of the ‘good life’ and, hence, personal life aspirations, as well as (2) their knowledge, awareness and perception of opportunities ‘here’ and ‘there’.

This paper has argued that we can achieve a more meaningful understanding of agency in migration processes by conceptualising migration as a function of aspirations and capabilities to migrate within given sets of perceived opportunity structures. On this basis, we can define human mobility as *people’s capability (freedom) to choose where to live* – including the option to stay – rather than the act of moving itself. Moving and staying then become complementary manifestations of the same migratory agency. This conceptualisation enables us to move beyond the futile debate over whether migration or sedentary behaviour is the norm, since a truly agentic view on migration does not presume either moving or staying as the norm, but acknowledges that they are two sides of the same freedom-of-mobility coin. This enables us to overcome dichotomous and simplistic classifications such as between forced and voluntary migration and to integrate the analysis of most forms of migratory mobility within one meta-conceptual framework.

The application to migration studies of Sen’s (1999) capabilities perspective on development creates the conceptual space to achieve a deeper understanding of the role of capabilities in shaping migration aspirations as well as to make a vital analytical distinction between the *instrumental* and the *intrinsic* dimensions of human mobility. In order to acquire a more systematic understanding of the dialectics between structure and agency in migration processes, Berlin’s (1969) distinction between positive and negative liberties is a useful theoretical tool to elaborate a more structured, systematic and contextualised view of how macro-structural change processes affect people’s aspirations *and* capabilities to migrate in complex, non-linear and frequently counter-intuitive ways.

The resulting framework creates significant scope for improved theoretical synthesis by integrating different migration theories under one meta-conceptual umbrella. Instead of being mutually exclusive, within this alternative vision different migration theories have various degrees of explanatory power to understand various forms of migration occurring under specific conditions, among particular social groups and migrant categories and at various levels of analysis. This exemplifies the broader argument of this paper, which posits that, in migration theory specifically and social theory more generally, theoretical assumptions should be seen as contextualised statements rather than as mutually exclusive truth claims.

While this paper is hopefully useful in the much-needed effort to elaborate a more comprehensive, contextualised and integrated theorisation of human mobility, significant additional conceptual work remains to be done. For instance, considerable theoretical progress can be achieved by further embedding migration studies within broader theories of social change. This can, for instance, be done through applying insights from fields such as social psychology and behavioural economics. Research in such fields has yielded advanced insights into people’s (often non-rational) behaviour and factors that may affect migration aspirations but have rarely been applied to migration studies. Significant progress can also be achieved by integrating social-scientific and historicising approaches to studying migration. While social-scientific perspectives on migration could benefit from a better historisation and the adoption of *longue durée* perspectives, historical studies of migration could benefit from the adoption of social-scientific perspectives and theories to elaborate improved explanatory accounts of historical migration trends – for instance through adopting historical comparative methods (see Vezzoli 2015).^6^ More generally, as the world and social realities are constantly changing, no social theory will ever be ‘final’, as ongoing processes of social transformation will perpetually create the need for theoretical innovation in order to make sense of these changing realities and the diverse ways in which people give meaning to them.

Future theoretical work can help us to address several remaining conceptual puzzles. For instance, we may wonder to what extent we can really separate intrinsic from instrumental migration aspirations, as they often seem conflated in practice. After all, what appears to be an intrinsic and subjective desire for adventure and the discovery of new horizons could, at least subconsciously, also fulfil a ‘functional’ role in the psychological separation-individuation process of adolescents and young adults, as a way to acquire new knowledge, meet future partners, find a job and establish independence. Conversely, what appears to be a move abroad to earn more money can be difficult to separate from the social prestige which successful migration – particularly when it initially involves significant risks and courage – can bring, particularly in communities where migration has become a *rite de passage* and is cast in positive cultural-normative ways.

Another, related conceptual puzzle is that of voluntariness. To what extent can we classify migration as voluntary if a migrant does not want to move but does so for the sake of the long-term economic future of the family? Perhaps we can argue here that such a migrant has no intrinsic desire to move but that the decision to move still emanates from an autonomous decision and real willingness to sacrifice short- to medium-term individual wellbeing (for instance, being separated from loved ones or being deprived of sexual relations or the alienating experiences of living in a strange and sometimes hostile society) from the (instrumental) wish to improve the long-term wellbeing of the family (presumably after return or family reunion). However, what if family members are put under immense social pressure to migrate against their own intrinsic desire? This could for instance apply to labour migrants who move abroad to work because of their family’s social expectations, although they may personally resent this; but it can also apply to adolescents sent to boarding school abroad by their affluent parents. What, therefore, can we say about children and adolescents who often have little input into the mobility decisions of their parents and who may feel that they are ‘moved around’ as if they were a piece of luggage. We cannot ignore the considerable emotional stress and social costs implicated in the loss of friends, a familiar environment, alienation and the constant need to adapt to new situations, even if they live a life of material privilege. This begs the question as to whether we can conceptualise such children as forced migrants.

This highlights the inherently blurred lines between the concepts of ‘voluntary’ and ‘forced’ migration. There are parallels with similar debates, such as those on the difficulties in conceptualising forced versus voluntary marriage (see Enright 2009). In such situations there is often a conflict between the desire to be a member of social groups for psychological and social-security reasons on the one hand and the personal drive towards autonomy on the other. This shows the importance of developing conceptual tools that can help us to gain more nuanced understandings of the interaction between structure and agency in social action. This also suggests that the aspirations–capabilities framework developed in this paper can be useful for other domains of social theory.

## Data Availability

This paper does not use any primary research data, and is fully based on other publications and the author’s analysis.

## Footnotes

1 This paper is not the appropriate place to extensively discuss the pros and cons of these different concepts, which all refer to social change but have different foci and disciplinary origins. For the sake of brevity, this paper will use the terms social transformation and development as shorthands to indicate broader, more fundamental processes of social change (for a more elaborate discussion, see de Haas et al. 2020b).

2 This also exemplifies the fundamentally problematic nature of transposing natural-science notions of causality to the social sciences, in both theorising and empirical analysis.

3 Economists would rather call this ‘human capital’.

4 In urbanized societies, and later in the lifecycle when people establish families, urbanites may aspire to live in the countryside again in search for calm, nature and community, or find a compromise by moving to suburbs.

5 I thank Sarah Salehi for bringing my attention to this point.

6 From this perspective, there is, indeed, good reason to consider history (also) as a social science.
